# Role of Quenching Temperature Selection in the Improvement of the Abrasive (Al_2_O_3_) Wear Resistance of Hybrid Multi-Component Cast Irons

**DOI:** 10.3390/ma17153742

**Published:** 2024-07-28

**Authors:** Yuliia Chabak, Vasily Efremenko, Ivan Petryshynets, Michail Golinskyi, Kazumichi Shimizu, Bohdan Efremenko, Vadim Kudin, Alexander Azarkhov

**Affiliations:** 1 Physics Department, Pryazovskyi State Technical University, 49044 Dnipro, Ukraine; chabak_y_g@pstu.edu (Y.C.); michalholynskyi99@gmail.com (M.G.); efremenko_b_v@pstu.edu (B.E.); 2 Division of Metallic Systems, Institute of Materials Research, Slovak Academy of Sciences, 04001 Kosice, Slovakia; ipetryshynets@saske.sk; 3 Graduate School of Engineering, Muroran Institute of Technology, 27-1 Mizumoto, Muroran City 050-8585, Japan; shimizu@muroran-it.ac.jp; 4 Biomedical Engineering Department, Pryazovskyi State Technical University, 49044 Dnipro, Ukraine; azarhov_a_y@pstu.edu; 5 Department of Machines and Technologies of Foundry Production, National University Zaporizhzhia Polytechnic, 69063 Zaporizhzhia, Ukraine; vadim.kudin1973@gmail.com

**Keywords:** multi-component cast iron, high boron content, quenching, carboborides, matrix, hardness, abrasive wear resistance

## Abstract

In this paper, enhancing the tribological characteristics of novel cast metallic materials—hybrid multi-component cast irons—by applying a strengthening heat treatment is described. The experimental materials were the cast alloys of a nominal composition (5 wt.% W, 5 wt.% Mo, 5 wt.% V, 10 wt.% Cr, 2.5 wt.% Ti, Fe is a balance) supplemented with 0.3–1.1 wt.% C and 1.5–2.5 wt.% B (total of nine alloys). The heat treatment was oil-quenching followed by 200 °C tempering. The quench temperature (QT) varied in the range of 900–1200 °C, with a step of 50 °C (with a 2-h holding at QT). The correlation of the QT with microstructure and properties was estimated using microstructure/worn surface characterization, differential scanning calorimetry, hardness measurement, and three-body-abrasive wear testing (using Al_2_O_3_ particles). The as-cast alloys had a multi-phase structure consisting of primary and/or eutectic borocarbide M_2_(B,C)_5_, carboborides M(C,B), M_7_(C,B)_3_, M_3_(C,B), and the matrix (ferrite, martensite, pearlite/bainite) in different combinations and volume fractions. Generally, the increase in the quenching temperature resulted in a gradual increase in hardness (maximally to 66–67 HRC) and a decrease in the wear rate in most alloys. This was due to the change in the phase-structure state of the alloys under quenching, namely, the secondary carboboride precipitation, and replacing ferrite and pearlite/bainite with martensite. The wear rate was found to be inversely proportional to bulk hardness. The maximum wear resistance was attributed to QT = 1150–1200 °C, when the wear rate of the alloys was lowered by three to six times as compared to the as-cast state. With the QT increase, the difference in the wear rate of the alloys decreased by three times. The highest abrasive resistance was attributed to the alloys with 1.1 wt.% C, which had a 2.36–3.20 times lower wear rate as compared with that of the reference alloy (13 wt.% Cr cast iron, hardness of 66 HRC). The effects of carbon and boron on hardness and wear behavior are analyzed using the regression models developed according to the factorial design procedure. The wear mechanisms are discussed based on worn surface characterization.

## 1. Introduction

White cast irons alloyed with carbide-forming elements (Cr, V, Mo, etc.) are used for a wide range of industrial applications where the abrasive, erosion, and corrosion-abrasive wear mechanisms crucially affect the lifespan of the machine parts and components [1,2]. These materials present a composite-like structure comprising hard carbide precipitates distributed within the tougher metal matrix; such a structure is most beneficial to resisting abrasive particles’ cutting or impact actions [3,4]. The chemical composition and structure of high-alloyed cast irons are tailored to meet the specific working conditions. The multi-component cast irons (MCCIs) were primarily developed for the rolls of hot rolling and pulverizing mills [5]. Following the concept of high-speed steel M2 (ASTM A600) [6], MCCIs include several carbide-forming elements (W, Mo, V, Cr, Nb) in nearly equal proportions (from 2–5 wt.% [7] to 5–10 wt.% [8]), which results in a multi-phase solidification with the formation of different eutectic carbides (M_2_C, M_6_C, MC, M_7_C_3_) [9,10]. MCCIs differ from M2 steel by a two-fold-increased carbon content (1.8–2.2 wt.%), allowing them to have a higher volume fraction of the carbide phase and to be used as cast materials [11]. According to Matsubara and co-workers [8,12,13] and other researchers [14,15,16,17], a combination of several types of carbides and matrixes (martensite, austenite) in alloyed white cast iron ensures its advanced abrasion wear resistance. Furthermore, the authors [18] repeatedly informed about an increased high-temperature erosion resistance of MCCIs enhanced by Co or Ni additions. The study of the multi-component high-Cr and Ni-hard-type cast irons supplemented with different combinations of strong carbide-forming elements (V-Nb-Ti-Cr [19], V-Nb-Ta [20], Zr-B [21]) allowed for deducing the positive effect of combining different types of carbide (MC, M_3_C, M_7_C_3,_ Cr5B3) and intermetallic (Fe_2_Zr, FeCrZr) phases to increase the mechanical properties and wear resistance of the alloys.

Heat treatment (HT) is usually applied to advance the exploitation properties of high-alloyed cast irons [22,23]. In major cases, the highest wear resistance is achieved after the “Quenching + Tempering” heat treatment. During holding under the quenching temperature, dispersed secondary carbides precipitate from austenite, which destabilizes the austenite-to-martensite transformation under cooling (the so-called “destabilization” process) [24,25], leading to a drastic hardness increase. For the cast irons of different alloying systems (high-Cr, MCCIs, Ni-hard, etc.), the optimal destabilizing temperature is in the range of 900–1100 °C [26,27,28]. Tempering is carried out either at low temperatures (200–300 °C) to relieve quenching stresses or at 500–600 °C to eliminate retained austenite (RA) through secondary carbides precipitation and martensite transformation (the secondary hardening) [29,30,31].

In order to meet the working conditions of the hot rolling mills, MCCIs are advised to be quenched from 1050 to 1100 °C and tempered at 500–550 °C [5]. Quenching leads to the precipitation of the secondary carbides MC, M_6_C, and M_7_C_3_ and the formation of a “martensite + RA” matrix where the RA fraction may reach 25 vol.% [32,33]. Under the tempering, the decomposition of RA and martensite takes place via the carbide precipitation. The RA decomposition plays the main role, leading to a sharp decrease in the RA volume fraction and a corresponding hardness increase to 900–940 HV_30_ [5,32,33]. The martensite decomposition may pass through the sequent carbide transformations accompanied by the carbide enrichment with the alloying element. Inthidech and Matsubara [34] illustrated this process for Mo-rich martensite as follows: martensite (Fe,Mo,C) → (Fe,Mo)_3_C → (Mo,Fe)_3_C → Mo_6_C. The structure formation in MCCIs under heat treatment is dependent on HT parameters and the chemical composition of the alloy. As follows from [5], the RA volume fraction in as-quenched MCCIs is increased with the quenching temperature and total carbon concentration. It was deduced [34] that the maximum MCCI hardness after heat treatment depends on the total carbon content and carbon phase distribution. This dependence is described by the C_bal_ parameter, which is calculated as C_bal_ = C − C_stoich_, where C_bal_ is the carbon fraction dissolved in the matrix, C is the total carbon content in the alloy (wt.%), and C_stoich_ is the carbon fraction consumed to form the carbides according to their stoichiometry. It was concluded [34] that the maximum MCCIs’ hardness after heat treatment is associated with a C_bal_ value close to zero %.

The secondary hardening behavior of MCCIs is found to be controlled by alloying elements. Thus, chromium (up to 5–7 wt.%) promotes the secondary carbide precipitation. With its further content increase, austenite stabilizes to phase-structural transformation (including the deformation-induced martensite transformation [15]) and secondary hardening decreases [34]; therefore, the maximum tempered hardness of MCCIs refers to 5–7 wt.% Cr [32]. A similar effect of nickel was noted by Zhang et al. in [18], where nickel was highlighted to stimulate the precipitation of secondary carbides in MCCIs due to the reduced solubility of carbon in austenite. The excessive addition of Ni significantly increases the content of retained austenite in quenched white cast iron [35], although it benefits the corrosion resistance of Fe-based alloys [36].

The MCCIs’ family includes newly developed hybrid multi-component cast irons, which are distinguished by the presence of both carbon and boron in the chemical composition [37,38,39]. Replacing carbon with boron makes it possible to form the complex-alloyed carboboride phases, which are harder than the carbides formed by the same carbide-forming elements [40,41]. In previous efforts of the authors [42], the influence of carbon and boron on the abrasive wear resistance of hybrid MCCIs in the as-cast state was studied based on the factorial design method. It has been found that their wear resistance varies significantly, being controlled by the carboboride particles’ features (type, size, distribution, etc.) and the structure of the metallic matrix. At the same time, there is a significant potential to increase the hybrid MCCIs’ wear resistance through heat treatment. Since the effect of HT parameters on the structure and properties of hybrid cast irons remained unstudied, this predetermined the performance of the present research.

Recently, the MCCIs’ family was expanded by adding hybrid multi-component (W, Mo, V, Cr, Ti) cast irons newly developed by the authors of the present work [37]. These alloys are distinguished by partially replacing carbon with higher amounts of boron (1.5–3.5 wt.%) [37,38,39]. The substitution of carbon with boron allows for the formation of the complex-alloyed carboboride phases [40,41,42], which are harder than the carbides formed by the same carbide-forming elements [43,44]. In fact, the use of boron as the main alloying element in wear-resistant high-boron alloys is well known [45,46]. However, the creation of a multiphase state comprising complex-alloyed carboboride phases of various types was realized in hybrid phases for the first time. In previous efforts of the authors [47], the influence of carbon and boron on the abrasive wear resistance of hybrid MCCIs in the as-cast state was studied based on the factorial design method. It was found that their wear resistance varied significantly being controlled by the carboboride particles’ features (type, size, distribution, etc.) and the structure of the metallic matrix. At the same time, there is a significant potential to increase the hybrid MCCIs’ wear resistance through heat treatment. This conclusion follows from the works [48,49] dedicated to the effect of heat treatment on the wear resistance of high-boron alloys of Fe-C-B, Fe-C-B-Cr, and Fe-C-B-Cr-Mo systems. In these articles, the process of the precipitation of the secondary carboborides Fe_2_B, (Fe,Cr)_2_B, (Cr,Fe)_23_(B,C)_6_, and boride M_3_B_2_ during high-temperature holding followed by martensite transformation is revealed as the crucial factor leading to a drastic increase in the hardness and abrasive wear resistance of high-boron alloys. However, the structure/properties evolution under high-temperature treatment in cast high-boron alloys with a more complicated chemical composition has not been well studied yet. In this case, the character of the influence of the heat treatment mode may change due to complex alloying with elements (W, Mo, Mn, Ti, etc.) affecting the solidification sequence and kinetics of phase transformations [50]. The lack of such data regarding hybrid multi-component alloys predetermined the performance of the present research. It was aimed at advancing the abrasive wear resistance of hybrid multi-component Fe-B-C-W-Mo-V-Cr-Ti alloys by applying the “Quenching and Low-temperature Tempering” heat treatment, with an emphasis on studying the effect of the quenching temperature on the structure evolution and the “Microstructure-Hardness-Wear resistance” correlations.

## 2. Materials and Methods

The research materials were the cast multi-component Fe-based alloys, with the chemical composition selected according to the matrix of factorial design of experiment 3^2^ (with two variables that were varied at three levels) [51,52] (Table 1). Each alloy nominally contained 5 wt.% W, 5 wt.% Mo, 5 wt.% V, 10 wt.% Cr, 2.5 wt.% Ti, 1 wt.% Mn, and 1 wt.% Si (Fe is a balance). The variable parameters were the carbon (X_1_) and boron (X_2_) contents. Carbon was changed at three nominal levels—specifically, 0.3 wt.% (lower), 0.7 wt.% (middle), and 1.1 wt.% (upper). At each of them, the boron content was varied at three levels: 1.5 wt.% (lower), 2.5 wt.% (middle), and 3.5 wt.% (upper). The levels were coded as (–1), (0), and (1) respectively. Thus, a total of nine alloys were fabricated as described in [37,38,39], with the actual chemical compositions presented in Table 1. The specimens of 6 × 12 × 25 (mm) in size were prepared by cutting from the cast ingots for further testing and structure characterization.

To improve the properties of alloyed cast irons, heat treatment is most often used according to the “destabilization” scheme, which includes a holding at the quenching temperature necessary for the secondary carbide precipitation [24]. The depleted austenite is easily transformed into martensite, resulting in hardness and wear resistance increases [53]. The same approach was adopted in this work. Since there are currently no data on the optimal quenching temperature (QT) for hybrid multi-component alloys, preliminary studies were carried out using differential scanning calorimetry (DSC). The phase transformation under heating was evaluated using the DTA-DSC-TG thermal analyzer “Jupiter STA449-F1” (Netzsch, Waldkraiburg, Germany). The scanning was performed in the temperature range of 20–1200 °C with a heating rate of 10 K·min^−1^; an empty crucible (Al_2_O_3_) was used as a reference. The “Netzsch Proteus Thermal Analysis 5.2.0” software was used to derive the transition temperatures from the DSC curves.

**Figure 1:**
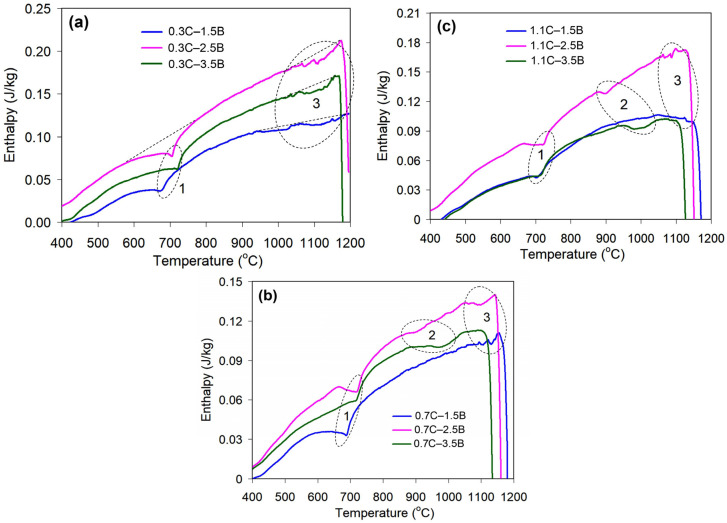
DSC curves of the alloys with different carbon contents: (**a**) 0.3 wt.%, (**b**) 0.7 wt.%, (**c**) 1.1 wt.%. (1—the “low-temperature” effects, 2—the “middle-temperature” effects, 3—the “high-temperature” effects).

The heating curves of the alloys are shown in Figure 1, and the results of their processing are presented in Table 2. Several endothermic effects are observed on each curve. All alloys performed a “low-temperature” effect (#1) in a temperature range of ~600–800 °C. The “middle-temperature” effect (#2) was revealed on the curves of the alloys containing 0.7–1.1 wt.% C and 2.5–3.5 wt.% B (Figure 1b,c) at a temperature range of ~880–1050 °C. The “high-temperature” effects (#3) were found for most alloys (except 1.1C–1.5B and 1.1C–3.5B) at ~980–1170 °C, close to the melting point (Figure 1a). At higher temperatures, DCS curves sharply decline, reflecting the onset of melting [54,55]. The “low-temperature” effect coincides with the temperature range of αFe → γFe transformation in Fe-C alloys (Ac_1_ temperature, 723 °C [56]). Therefore, the quenching temperature (QT) range was selected to be higher than the “low-temperature” effects, pursuing the formation of austenite as a prerequisite for austenite → martensite transformation during quenching cooling. The QTs range was set as 950–1200 °C with a step of 50 °C (the temperatures of 1150 °C and 1200 °C were selected close to the melting point to modify the shape of the carboboride particles by diffusion simulation). The holding at a quenching temperature (2 h) was sufficient for the secondary particle precipitation completion [26]. The heating was performed in a muffle furnace in a protective nitrogen atmosphere. The heated specimens were oil-cooled and tempered at 200 °C for 2 h. Before the hardness and wear testing, the heat-treated specimens were ground to the roughness of Ra ~0.6 μm, removing the oxidized layer of 0.5 mm thickness. 

The microstructure was studied using the specimens prepared by the routine procedure of a surface mirror-polishing and etching with 5 vol.% Nital solution. The microstructure was observed by employing the optical microscopes (OM) “Eclipse M200” (Nikon, Tokyo, Japan) and “GX71” (Olympus, Tokyo, Japan) and the scanning electron microscope (SEM) “JSM-7000F” (JEOL, Tokyo, Japan). The phase constituents were identified by X-ray diffraction using a diffractometer “X’Pert PRO” (PANalytical, Worcestershire, UK) with Cu-Kα radiation under the following parameters: the voltage is 40 kV, the tube current is 50 mA, the scan step is 0.033 degrees, and the scan speed is 0.069 degree·s^−^^1^. The “FR-X1” tester (Future-Tech, Kanagawa, Japan) was used to measure the bulk hardness of the alloys according to the Rockwell method (scale C). Fifteen measurements were made on each alloy/QT with further results averaging.

The “Three-Body Abrasion” scheme was used for the wear performance assessment. Under the test, the specimen was pressed (with a force of 20 N) to a 40 mm diameter rubber roller rotating at 650 rpm. The abrasive material (Al_2_O_3_ particles of ~0.5 mm diameter) was fed between the roller and the specimen’s surface with a feed rate of 0.75 kg·min^−1^. The test duration was 1800 s. The weight loss of the specimen was measured using an electronic balance of 0.0001 g accuracy. Each test was repeated three times to average the results. The wear resistance was estimated by the wear rate (*WR*), calculated as:

(1) $$WR=\frac{\Delta m}{b\cdot t}$$

where *b* stands for the specimen width (mm) and *t* stands for the test duration (s).

The data on the bulk hardness and wear rate of as-cast hybrid MCCIs were adopted from our previous work [39] to reveal the effect of heat treatment on the alloys’ properties. The studied alloys were compared with a high-Cr cast iron which was used as a reference alloy (with a chemical composition of 3.2 wt.% C, 1.2 wt.% Si, 1.5 wt.% Mn, 13.2 wt.% Cr). After destabilizing heat treatment (QT of 950 °C, tempering at 200 °C), the reference had a bulk hardness of 66 HRC.

## 3. Results and Discussion

### 3.1. As-Cast Structure of Studied Alloys

**Figure 2:**
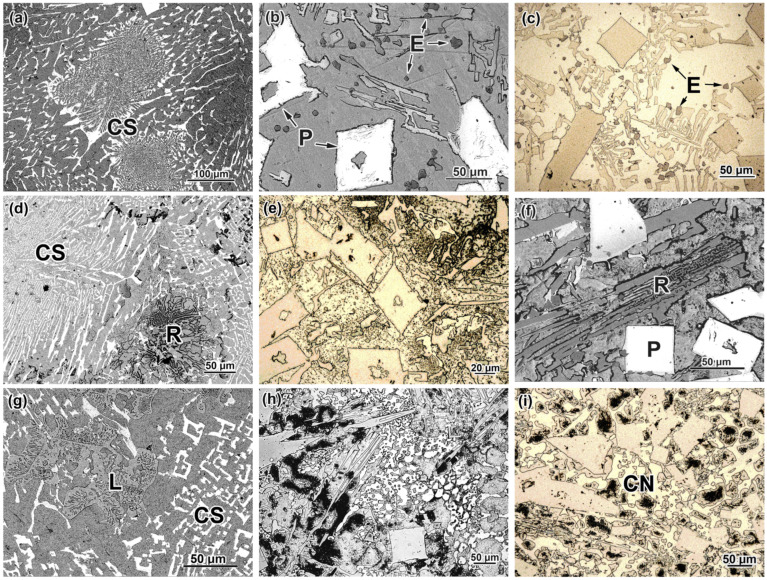
Microstructure of as-cast hybrid MCCIs: (**a**) 0.3C–1.5B, (**b**) 0.3C–2.5B, (**c**) 0.3C–3.5B, (**d**) 0.7C–1.5B, (**e**) 0.7C–2.5B, (**f**) 0.7C–3.5B, (**g**) 1.1C–1.5B, (**h**) 1.1C–2.5B, (**i**) 1.1C–3.5B (CS is a “Chinese-script” eutectic; P is a primary carboboride M_2_(B,C)_5_; R is a “Rosette” eutectic; CN is a “Coarse-net” eutectic; L is a Ledeburite eutectic; E is an equiaxed particle M(C,B)). ((**a**,**b**,**d**,**f**,**g**)—SEM/BSE images; (**c**,**e**,**h**,**i**)—OM images).

In this subsection, the initial (as-cast) microstructure of the experimental MCCIs is briefly described based on our articles [37,38,39] which present the comprehensive structural characterization of these alloys. The structure images of the hybrid MCCIs are depicted in Figure 2. In each group of alloys with a constant carbon content, the following tendency was noted: the alloy with 1.5 wt.% B had a nearly eutectic structure, while the alloys with 2.5–3.5 wt.% B belonged to a hypereutectic domain. In low-boron alloys (1.5 wt.% B), an eutectic of a “Chinese-script” (CS) morphology was the major structural constituent, consisting of long thin fibers branched within the matrix (Figure 2a). These fibers are a (W,Mo,V)-rich borocarbide M_2_(B,C)_5_ which has a light contrast on the back-scattered electron (BSE) images due to enrichment by elements with high atomic numbers (W, Mo). When the carbon content increases to 0.7–1.1 wt.%, another eutectic appears in a minor amount—specifically, (a) a “Rosette”-like (R) eutectic based on the Cr-rich carboboride M_7_(C,B)_3_ (in alloy 0.7C–1.5B, Figure 2d) or (b) the “Ledeburite”-type eutectic based on the Fe-rich boron-cementite M_3_(C,B) (in alloy 1.1C–1.5B, Figure 2g).

In hypereutectic alloys (2.5–3.5 wt.% B), the primary boride M_2_(B,C)_5_ was present in the form of prismatic-shaped coarse particles with the dimensions of tens of micrometers. These particles were surrounded by the colonies of the eutectics of different types. In the alloys containing 2.5 wt.% B, the major eutectic constituent was a CS, while the minor constituent was an R-eutectic (alloy 0.7–2.5B, Figure 2e) or two eutectics: an R-eutectic and an M_3_(C,B)-based “Coarse-net” eutectic in the form of a thick continuous network engulfing the matrix grains (alloy 1.1C–2.5B, Figure 2h). In the alloys containing 3.5 wt.% B, a “Chinese-script” eutectic was absent. Instead, an R-eutectic (at 0.3–0.7 wt.% C, Figure 2c,f) or a “Coarse-net” eutectic (at 1.1 wt.% C, Figure 2i) were present.

Apart from the above-noted structural constituents, the small-sized angular equiaxed particles of Ti-based carboboride M(C,B) (shown in Figure 2b,c) were dispersed in all alloys. They are seen in BSE images as dark precipitates due to the presence of 55–73 wt.% of Ti having a lower atomic number (relative to Fe and other alloying elements).

The metallic matrix of the alloys containing 0.3 wt.% C (at any boron content) and the alloys containing 1.5 wt.% B (at 0.7–1.1 wt.% C) was ferrite. With further carbon and boron content increases, the matrix transformed to martensite (at 2.5 wt.% B) or to a “martensite + lamellar conglomerate (with morphological attributes of pearlite or upper bainite)” (at 3.5 wt.% B). In the matrix of the alloy 1.1C–3.5B, a minor amount of ferrite was also present. Moreover, retained austenite was present in a minor amount (6–15 vol.%) in the structure of all alloys [39].

### 3.2. Effect of Quenching Temperature on Bulk Hardness

**Figure 3:**
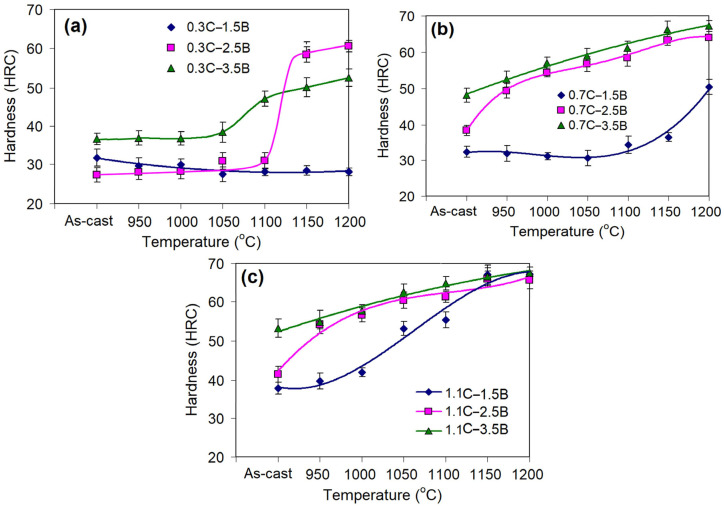
Effect of quenching temperature on the bulk hardness of MCCIs containing (**a**) 1.5 wt.% B, (**b**) 2.5 wt.% B, and (**c**) 3.5 wt.%.

The evolution of the bulk hardness of the alloys is illustrated in Figure 3. In the as-cast state, the hardness varied in the range of 28–53 HRC; it increased with the boron and carbon contents. Specifically, for the alloys containing 0.3 wt.% C, the hardness varied in the range of 28–37 HRC. In alloys with 0.7 wt.%, the hardness was higher—32–48 HRC [39]. The maximum hardness diapason (38–53 HRC) was attributed to the alloys having 1.1 wt.% C. This tendency was due to an increase in the volume fraction of hard phase precipitates (borocarbides, carboborides) caused by the addition of carbon and boron [39]. Also, the hardness was affected by the type of matrix: the lowest hardness values were associated with the ferritic matrix, while the higher hardness referred to the martensite of “martensite + pearlite (bainite)” matrixes.

Heat treatment resulted in a significant increase in the bulk hardness of most alloys, while the hardness profile depended on the alloys’ chemical composition. In all alloys containing 0.3 wt.% C and/or 1.5 wt.% B (at any other C/B contents), the hardness was increased only at QT ≥ 1100 °C. In contrast, in alloys with 0.7–1.1 wt.% C and 2.5–3.5 wt.% B, the hardness grew gradually within the entire QT interval. More specifically, the alloys with 0.3 wt.% C and alloy 0.7C–1.5B hardly changed their hardness when the QT was within 950–1050 °C (Figure 3a,b). After quenching from higher temperatures, the hardness was doubled (to 60 HRC) in the alloy 0.3C–2.5B and increased by 1.5 times (to 50 HRC) in the alloy 0.7C–1.5B. The alloy 0.3C–1.5B was not affected by quenching; its hardness varied in the range of 28–32 HRC at any QT.

In other alloys, the hardness gradually increased with a QT increase. The most significant hardness improvement was observed in the alloys 0.7C–2.5B and 1.1C–2.5B: their hardness rose from 38–39 HRC to 63 HRC and 68 HRC, respectively (Figure 3b,c). The alloys with a maximum B content (0.7C–3.5B and 1.1C–3.5B) gained the same high hardness of >65 HRC. The alloys that had the martensite or “martensite + pearlite (bainite)” matrixes in an as-cast state were more sensitive to quenching, while the alloys with a ferrite matrix reacted to quenching only at high QTs.

### 3.3. Effect of the Quenching Temperature on the Wear Rate

**Figure 4:**
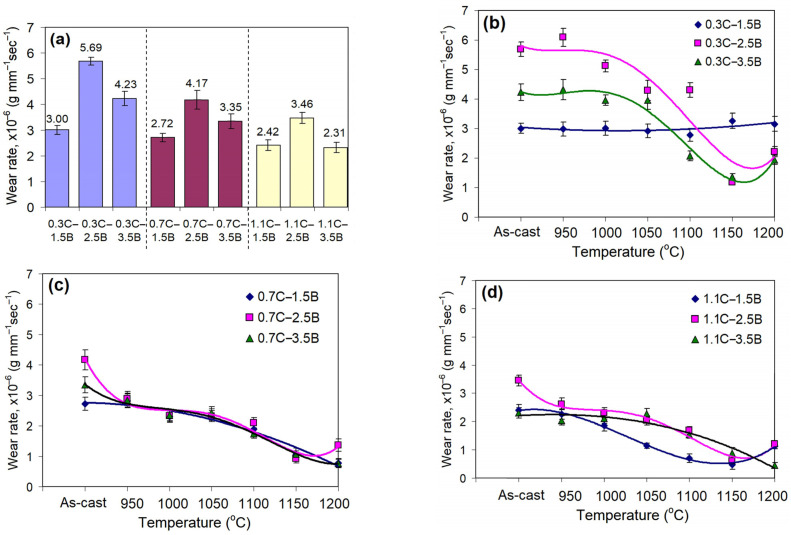
(**a**) *WR* values of the as-cast alloys. Effect of quenching temperature on the wear rate of the alloys containing (**b**) 0.3 wt.% C, (**c**) 0.7 wt.% C, and (**d**) 1.1 wt.% C.

Figure 4 presents the MCCIs’ wear rate values in the as-cast state (Figure 4a) and depending on the QT (Figure 4b–d). In the as-cast state, the MCCIs’ wear rate decreased with an increase in the carbon and boron contents [41] as follows: the alloys with 0.3 wt.% C (*WR* of (3.0–5.69) × 10^−6^ g·mm^−1^·s^−1^) → 0.7 wt.% C (*WR* of (2.72–4.17) × 10^−6^ g·mm^−1^·s^−1^) → 1.1 wt.% C (*WR* of (2.31–3.46) × 10^−6^ g·mm^−1^·s^−1^) (Figure 4a). Under quenching, in each group, the *WR* values were decreased with a quenching temperature increase (the only exception was the alloy 0.3C–1.5B, which was not affected by quenching at any QT, as seen in Figure 4b). The alloys with 0.7–1.1 wt.% B demonstrated close *WR* values at different QTs (Figure 4c,d). Most alloys reached the minimum *WR* value after quenching from 1150 °C; at QT = 1200 °C, their *WR* values slightly increased. In the alloys 0.7C–1.5B, 0.7C–3.5B, and 1.1C–3.5B, the lowest *WR* values are ascribed to QT = 1200 °C. After quenching from 1150–1200 °C, the alloys with 0.7 wt.% C performed a wear rate less than 2.0 × 10^−6^ g·mm^−1^·s^−1^, while the alloys with 1.1 wt.% showed an even lower *WR* of ≤1.0 × 10^−6^ g·mm^−1^·s^−1^.

Assessing the overall picture, one can conclude that in the heat-treated state, the highest *WR* value was attributed to the alloys 0.3C–1.5B (6.09 × 10^−6^ g·mm^−1^·s^−1^, QT = 950 °C). Accordingly, the lowest *WR* values referred to the alloy 1.1C–1.5B (0.88 × 10^−6^ g·mm^−1^·s^−1^, QT = 1150 °C) and the alloy 1.1C–3.5B (0.81 × 10^−6^ g·mm^−1^·s^−1^, QT = 1200 °C). With a QT increase, the difference in the wear rate between the alloys decreased significantly (as compared with that in the as-cast state).

The decrease in the *WR* value relative to that in the as-cast state reflects the improvement in the wear resistance of the alloy caused by the heat treatment. To assess this improvement, the wear rate decrease index (*WRD*) was calculated for each alloy as follows:

(2) $$WRD=\frac{WR_{As–c}}{WR_{q}},$$

where *WR_As–c_* and *WR_q_* are the wear rates for the as-cast and quenched states, respectively.

**Figure 5:**
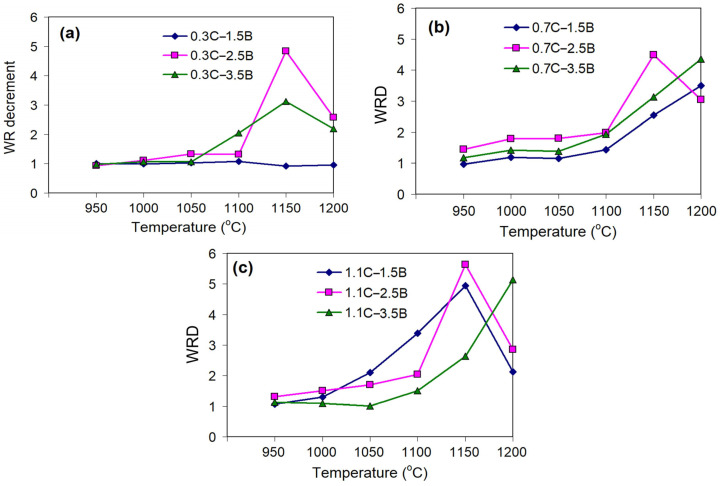
Effect of quenching temperature on the *WRD* in the alloys containing (**a**) 0.3 wt.% C, (**b**) 0.7 wt.% C, and (**c**) 1.1 wt.% C.

As follows from Figure 5, in general, the *WRD* increases with a QT increase, while its more noticeable growth refers to the quenching from higher temperatures (QT ≥ 1100 °C). Depending on the carbon content, the *WRD* reaches, maximally, 3.12–4.84 (at 0.3 wt.% C) (Figure 5a), 3.50–4.49 (at 0.7 wt.% C) (Figure 5b), and 4.49–5.63 (at 1.1 wt.% C) (Figure 5c), i.e., carbon enhances the effect of quenching on the wear resistance of hybrid MCCIs. Such a significant improvement in wear behavior relative to the as-cast state shows the potential of heat treatment to control the exploitation durability of these alloys. The *WRD* curves’ maximum was attributed to either QT = 1150 °C or QT = 1200 °C. The decrease in the *WRD* at 1200 °C was caused by the partial melting that occurred in the alloys 0.3C–2.5B, 0.3C–3.5B, 0.7C–2.5B, 1.1C–1.5B, and 1.1C–2.5B. In these alloys, the *WRD* decreased even despite the fact that the hardness reached a maximum value.

### 3.4. Effect of Quenching Temperature on Structure Evolution

As follows from Figure 3 and Figure 4, the bulk hardness and *WR* values were strongly dependent on the quenching temperature being a result of a corresponding structure evolution. Microstructure characterization reveals that in all alloys, the carboboride primary particles (both primary and eutectic) were hardly affected by heat treatment regarding their shape and volume fraction (even after heating at 1150 °C, the precipitates mostly remained in the initial state due to their high heat stability). The main structural changes occurred precisely in the metal matrix of the alloys, namely, (a) the precipitation of secondary carboboride phases in the form of dispersed, evenly distributed grainy precipitates and (b) the formation of an acicular martensite structure. In each alloy, these changes occurred starting from a specific QT. In the alloys with an initial ferritic matrix (0.3 wt.% C-containing alloys, 0.7C–1.5B and 1.1C–1.5B alloys), martensite appeared at a rather high QT which varied from 1050 °C (0.3C–3.5B and 1.1–1.5B alloys) to 1150–1200 °C (alloys 0.3C–2.5B and 0.7C–1.5B). In alloys that initially had a “martensite + pearlite (bainite)” matrix, the fully martensitic matrix was formed starting from QT = 950 °C. The alloy 0.3C–1.5B retained the ferritic matrix regardless of the quenching temperature.

**Figure 6:**
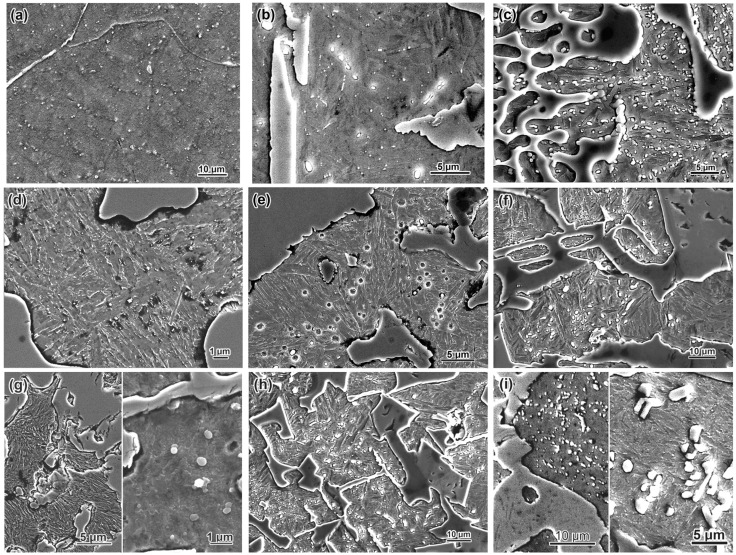
Microstructure of the alloys after the heat treatment with QT = 1150 °C: (**a**) 0.3C–1.5B, (**b**) 0.3C–2.5B, (**c**) 0.3C–3.5B, (**d**) 0.7C–1.5B, (**e**) 0.7C–2.5B, (**f**) 0.7C–3.5B, (**g**) 1.1C–1.5B, (**h**) 1.1C–2.5B, (**i**) 1.1C–3.5B.

**Figure 7:**
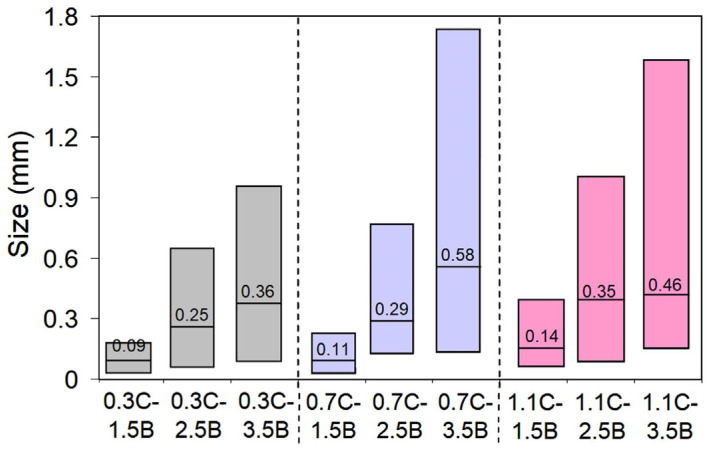
The size of secondary precipitates in the MCCIs after quenching. The line stands for a mean value; the rectangle shows the scatter of the values.

Figure 6 shows the microstructure of the metal matrix of the studied alloys after quenching from 1150 °C. In the alloys, except for 0.3C–1.5B, the matrix was a fine-needle martensite, in which the secondary carboboride precipitates were distributed. Within each group of alloys with the same carbon content, the number of secondary carboboride particles increased with the boron content. The lowest number of inclusions was observed in alloys containing 1.5 wt.% B (Figure 6a,d,g). According to Figure 7, the mean size of the secondary precipitates and the size scatter were increased with the C and B contents. The most dispersed inclusions were recorded in the alloy 0.3C–1.5B; their size varied within 0.01–0.15 μm. The higher scatter ranges were attributed to the alloys 0.7C–3.5B and 1.1C–3.5B, being 0.30–1.74 μm and 0.30–1.58 μm, respectively.

**Figure 8:**
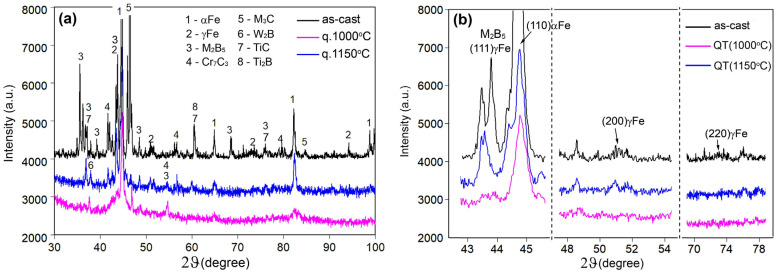
XRD patterns of the alloy 1.1C–3.5B: (**a**) a total 2θ range, (**b**) variation in the intensity of austenite peaks (111)_γ_, (200)_γ_, and (220)_γ_ depending on the alloy’s state (as-cast, after quenching).

The effect of the heat treatment on the variation of the phase composition of the alloys is illustrated in Figure 8, where the XRD patterns of the alloy 1.1C–3.5B are shown. As seen, the diffraction patterns for the as-cast and quenched specimens are similar, presenting various peaks of αFe, indicating its majority in the alloy matrix. There are also rather strong peaks of boride M_2_B_5_ (at 2θ = 35.6°) and carbide M_3_C (at 2θ = 46.2°) relating to the coarse primary inclusions M_2_B_5_ and the “Coarse-net” eutectic (Figure 8a). At 2θ of (43.5–43.8°), all curves contain a peak that can be attributed to both austenite ((111)_γ_) and M_2_B_5_ due to the similarity in the interplanar spacing. In addition, all diffraction patterns present multiple weak peaks which can be attributed to carbides (Cr_7_C_3_, M_3_C, TiC), borides (W_2_B, Ti_2_B), and austenite.

Figure 8b shows the enlarged sections of the diffraction patterns related to the austenite lines (111)_γ_, (200)_γ_, and (220)_γ_. It allows for following up the variation in the RA volume fraction depending on the quenching temperature. It is seen that from the as-cast state to 1150 °C-quenching, the intensity of all three peaks decreases. Specifically, the weak peaks (200)_γ_ and (220)_γ_ disappeared completely on the patterns, and the peak at 2θ = (43.5–43.8)° significantly reduces its intensity relative to the peak (110)α (moreover, this remaining weak peak may refer to boride M_2_B_5,_ which coincides with austenite at that 2θ). This indicates that after quenching from 1150 °C, the RA volume fraction decreased below the sensitivity level of the X-ray diffraction. It should be noted that on the XRD patterns of 1150 °C, no increase in the intensity of the carbide or boride lines was noticed, which could be caused by the secondary particles’ precipitation. At the same time, the peaks of an 1150 °C-quenched specimen were broadened, indicating increased residual stresses and lattice distortion.

### 3.5. Worn Surface Characterization

**Figure 9:**
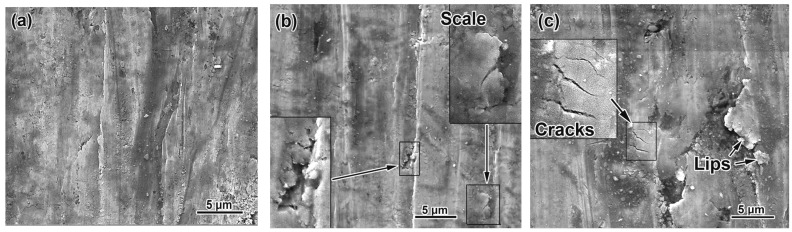

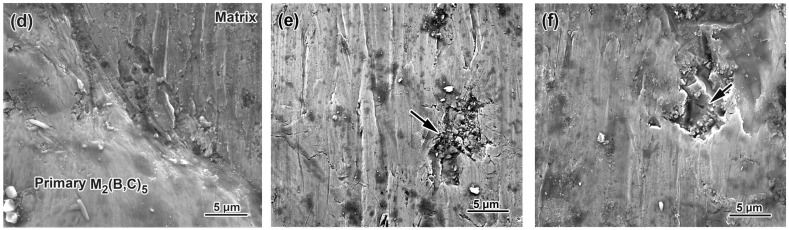
The patterns of the worn surfaces of the quenched alloys: (**a**) 1.1C–1.5B (QT = 1150 °C), (**b**) 0.7C–1.5B (QT = 1150 °C), (**c**) 0.3C–1.5B (QT = 1050 °C), (**d**) 0.3C–3.5B (QT = 1150 °C), (**e**) 0.7C–2.5B (QT = 1150 °C), (**f**) 1.1C–3.5B (QT = 1150 °C).

The worn surface of samples of various alloys were studied; the results are presented in Figure 9. In all cases, the surface of the samples was covered with grooves of different widths and depths. At high hardness, the grooves did not have clear boundaries which were smoothed and rounded (Figure 9a). As the hardness of the alloy decreased, the depth of the scratches increased, and ridges of extruded metal were observed along their edges (Figure 9b,c). In alloys with a eutectic structure (i.e., containing 1.5 wt.% B), the destruction of the surface proceeded through the fatigue-related process, resulting in the formation and detachment of wear debris in the form of metallic micro-scales (right insert in Figure 9b). The micro-scale was a volume of metal extruded from the groove and underwent repeated plastic deformation, which made it hard and brittle, to be easily broken off from the basis. In the alloy 0.3C–1.5B, which had low hardness at any quenching temperature, a larger volume of metal was involved in repeated deformation, which caused the appearance of larger flattened areas (the “lips” in Figure 9c), which were peeled off, increasing the wear rate. The bottom of the scratches also experienced strain hardening, which led to metal embrittlement and the appearance of micro-cracks oriented perpendicular to the abrasive sliding direction (insert in Figure 9c). In addition, micro-pits were occasionally observed on the surface of eutectic alloys, apparently caused by the destruction and by the eutectic carboboride breaking off (left insert in Figure 9b).

The worn surface of hypereutectic alloys (2.5–3.5 wt.% B) exhibited features similar to those of eutectic alloys (i.e., grooves, micro-scales, micro-cracks). However, they are distinguished by the presence of large pittings resulting from the spalling-off of coarse primary borocarbide M_2_(B,C)_5_ (locations are shown by arrows in Figure 9e,f). The pitting formation was preceded by the preferential wear of the matrix around the primary borocarbide (Figure 9d), resulting in the protrusion of borocarbide out of the matrix. Bending loads caused by sliding abrasive particles resulted in the cracking and spalling-off of the borocarbide from the surface.

**Figure 10:**
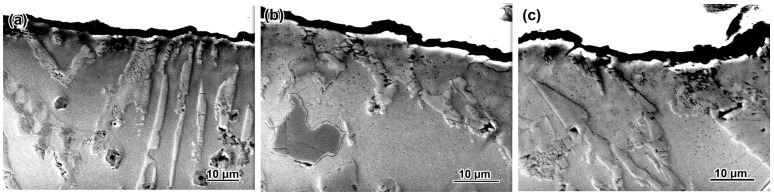

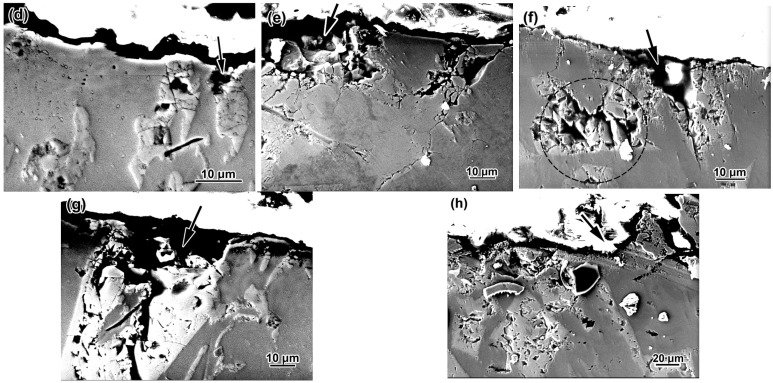
Cross-section of the worn surface of the alloys (QT in parentheses): (**a**) 0.3C–1.5B (1050 °C), (**b**) 0.7C–1.5B (1150 °C), (**c**) 1.1C–1.5B (1050 °C), (**d**) 1.1C–1.5B (11050 °C), (**e**) 0.3C–3.5B (1150 °C), (**f**) 0.7C–2.5B (1050 °C), (**g**) 0.7C–2.5B (1150 °C), (**h**) 1.1C–3.5B (1150 °C).

The wear mechanism is additionally illustrated by the cross-sectional images of the near-surface layer shown in Figure 10. In the eutectic alloys, the fibers of M_2_(B,C)_5_ (belonging to the “Chinese-script” eutectic) are bent following the matrix, which causes the micro-crack formation (Figure 10a–c). However, their bulk chipping was hardly observed; instead, an occasionally noticed breakage (shown by the arrow in Figure 10d) referred to the fracture of the more coarse eutectic carboborides M_7_(C,B)_3_ or M_3_(C,B), present along with the CS eutectic. The surface relief of the eutectic alloys varies depending on the bulk hardness from 5–6 µm to about 1 µm.

Regardless of the hardness, the hypereutectic alloys differed by the presence of deep (up to 15–20 µm) pits resulting from the spalling of the primary borocarbide (shown in Figure 10e–h by an arrow). In this case, not only inclusions on the surface but also those lying at some distance from it (shown by a circle in Figure 10f) were fractured. Hence, the roughness of the worn surface was increased relative to eutectic alloys. The destruction and spalling of the inclusions were accompanied by the cracks’ propagation from the surface inward of the specimen. Eutectic carboborides located next to the primary inclusion deteriorated without the pitting formation (Figure 10g).

## 4. Analysis of Results

### 4.1. “Quenching Temperature—Microstructure—Properties” Correlations

As shown above, quenching significantly enhances the hardness and wear resistance of MCCIs by changing a metallic matrix structure from ferrite or pearlite (bainite) to acicular martensite. Such structure evolution requires consecutive phase transformations, namely, αFe → γFe and γFe → αFe. On the DSC curves of all experimental alloys, an endothermic (“low-temperature”) effect was recorded at ~600–800 °C, which could be considered the αFe → γFe transition (an A_C1_ point) [56]. However, in some alloys (having 0.3 wt.% C, as well as alloy 0.7C–1.5B), an increase in hardness (connected with martensite formation) was found at a much higher QT (above 1000 °C). Moreover, the alloy 0.3C–1.5B remains in the ferritic state at any quenching temperature, meaning the absence of phase transitions. These results allowed for deducing that the “low-temperature” DCS effect did not refer to the A_C1_ temperature. More likely, it was associated with the Curie temperature transition, which is also accompanied by an endothermic effect [57,58]. Under a low carbon content (0.3 wt.%), the effect of ferrite-stabilizing elements (Cr, W, Mo, V) predominated under solidification, resulting in a fully ferritic matrix in the alloys 0.3C–1.5B, 0.3C–2.5B, and 0.3C–3.5B. This observation complies with a thermodynamic simulation [37], according to which the γ-phase domain was absent in the phase diagrams of the 0.3 wt.% C-MMCIs. Similarly, the alloys 0.7C–1.5B and 1.1C–1.5B also had a fully ferritic matrix owing to the enrichment of their matrix with Cr, W, Mo, and V [39].

The alloys with 0.7–1.1 wt.% C and 2.5–3.5 wt.% B, the coarse primary inclusions of M_2_(B,C)_5_, solidified, absorbing a significant portion of the alloying elements, thus leading to (Cr, W, Mo, V)-depletion of the matrix [37,38,39]. In combination with an increased concentration of carbon (gamma-stabilizer), this expanded the austenite domain, allowing for structure variation through austenite transformations. On the DSC curves of hypereutectic alloys, a fairly pronounced endothermic “middle temperature” effect (#2) was observed at ~900–970 °C (at 2.5 wt.% B) and ~900–1050 °C (at 3.5 wt.% B). These temperature ranges approximately coincide with the QT at which the hardness started to increase in these alloys. Therefore, it was assumed that DSC effect #2 is associated with the αFe → γFe transition leading to the austenite appearance in structure (with its further transformation into martensite upon quenching cooling). The temperature of αFe → γFe transformation in (0.7–1.1 wt.% C)-alloys significantly exceeds that in a grey cast iron (727 °C [59]) owing to an increased number of ferrite-stabilizing elements solved in the matrix (5–10 wt.% Cr, 0.7–1.0 wt.% Mo, 0.8–1.3 wt.% V [37]). Unlike this, in hypereutectic alloys with 0.3 wt.% C (0.3C–2.5B and 0.3C–3.5B), the αFe → γFe transition was shifted to even higher temperatures (~1020–1180 °C, DSC effect #3) because of the lower content of the austenite-forming element (which is carbon). Accordingly, an increase in their hardness was also noted at a higher QT (≥1050 °C).

The hardness and wear resistance of MCCIs were found to increase with the quenching temperature. The reasons are (a) reaching the γFe-domain under heating (which is situated rather high in MCCIs as compared to in plain cast iron) and (b) stimulating the secondary carboboride precipitation. With an increasing QT, more ferrite is substituted by austenite (resulting in an increase in the martensite fraction after cooling). Furthermore, the carbide precipitation proceeds more fully via diffusion stimulation; this promotes the austenite depletion in carbon and alloying elements to a greater extent, thus reducing the amount of retained austenite in the structure (accordingly, the proportion of martensite is also increased).

In the as-cast state, the alloys 0.7C–2.5B and 1.1C–2.5B had a completely martensitic matrix. Therefore, the significant increase in their hardness after quenching cannot be attributed only to a variation in the matrix status. Obviously, in this case, quenching caused a change in the martensite structure relative to the as-cast state, which promoted its hardness. A possible reason is the replacement of lath martensite with twinned martensite, caused by an increase in the carbon content and tetragonality [60]. Also, the hardness increase could be connected with the carboborides transformations, leading to changes in their structure and properties. These transformations can refer to the endothermic effects (#3) observed on the DSC curves at a high temperature range (Figure 1).

The formation of a martensitic matrix, strengthened by secondary carbides, is the main factor in increasing the hardness and abrasive wear resistance of the studied alloys. The heterogeneous composite-like structure is worn out unevenly, with preferential wear of the softer matrix and spalling-off of the naked hard inclusions. This mechanism is more likely for elongated particles oriented nearly perpendicular to the surface [61]. Long prismatic inclusions of primary borocarbide M_2_(B,C)_5_, being protruded over the surface, experience bending loads from sliding abrasive particles; this causes their cleavage into separate fragments (Figure 10e–h). After particle spalling-off, pitting appears, which accelerates the matrix wear by causing turbulence in the abrasive particle flow [62]. Thus, when abrasive particles cannot cut hard inclusions, the lifespan of the alloy is limited precisely by the matrix wear resistance. The appearance of martensite instead of ferrite or pearlite (bainite) stabilizes the wear process and slows down the destruction of the matrix. Eventually, borocarbide inclusions remain on the surface longer, performing a protective function and resulting in a sharp reduction in the wear rate. In an as-cast (soft) state, eutectic alloys have an advantage over hypereutectic alloys due to a more appropriate wear mechanism. When the matrix hardness increases, the difference in the wear resistance of the alloys is significantly reduced since the deteriorating effect of coarse primary borocarbides is diminished. Since MCCIs’ hardness strongly depends on the QT, the quenching temperature is a key factor determining their durability under abrasive wear conditions. The selection of the QT should pursue the achievement of the maximum hardness of the alloys, which will allow for fully using the advantage of high-hard carboboride inclusions formed in hybrid MCCIs due to complex alloying.

### 4.2. Effect of C and B Contents on Bulk Hardness and Wear Rate Depending on QT

The results of the bulk hardness measurements and abrasive wear tests were mathematically processed according to the procedure of a full factorial design method of 3^2^. This method allows for the construction of the model on the effect of two independent factors in the form of a fourth-degree regression equation, which can adequately describe a complex response surface [51,63]. The calculation approach is described in detail in our previous work [43]. In this section, the regression equations for the bulk hardness and wear rate after quenching from 1050 °C and 1150 °C were derived and analyzed (with an adequacy confirmation using the F-criterion). The equations were compared with the as-cast state, aiming to reveal the effect of C and B contents on the hardness and wear behavior depending on the quenching temperature.

After the calculations, the equations for bulk hardness were presented as follows:

*H_As-cast_* = 38.50 + 7.15*X*_1_ + 7.95*X*_2_ + 2.68*X*_1_*X*_2_ − 4.15*X*_1_^2^ + 1.75*X*_2_^2^ + 3.83*X*_1_^2^*X*_2_^2^ − 1.33*X*_1_*X*_2_^2^ − 2.88*X*_2_*X*_1_^2^, (3)

*H_1050_* = 56.70 + 14.70*X*_1_ +14.20*X*_2_ − 0.454*X*_1_*X*_2_ − 11.10*X*_1_^2^ − 11.90*X*_2_^2^ + 11.65*X*_1_^2^*X*_2_^2 −^ 2.25*X*_1_*X*_2_^2^ − 9.15*X*_2_*X*_1_^2^, (4)

*H_1150_* = 63.30 + 3.40*X*_1_ + 16.35*X*_2_ − 5.70*X*_1_*X*_2_ − 0.90*X*_1_^2^ − 13.45*X*_2_^2^ + 3.90*X*_1_^2^*X*_2_^2^ + 10.30*X*_1_*X*_2_^2^ − 10.90*X*_2_*X*_1_^2^, (5)

where *H* is the bulk hardness (HRC), and *X*_1_ and *X*_2_ are the coded levels of carbon and boron contents, respectively (see Table 1).

The free term of the Equations (3)–(5) increases from 38.5 (the as-cast state) to 63.3 (the 1150 °C-quenching), reflecting the general increase in bulk hardness under heat treatment. The positive values of the coefficients at *X*_1_ and *X*_2_ indicate that carbon and boron enhance the hardness of alloys. For the as-cast state and 1050 °C-quenching, the effect of carbon and boron on hardness was approximately the same. In the case of 1150 °C-quenching, the influence of boron was more significant (3.40*X*_1_ against 16.35*X*_2_ in Equation (5)).

**Figure 11:**
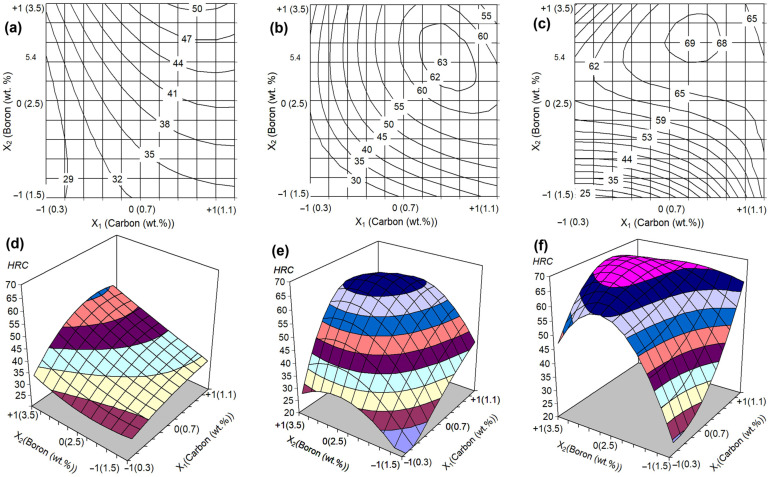
Graphical interpretation of the Equations (3)–(5). (**a**–**c**) The response surfaces of a bulk hardness and (**d**–**f**) its projections on the concentration plot. (**a**,**d**) The as-cast state, (**b**,**e**) QT = 1050 °C, (**c**,**f**) QT = 1150 °C.

As follows from the graphical interpretation of Equations (3)–(5) (Figure 11), in all three cases, both carbon and boron simultaneously increase the hardness of the alloys, resulting in a response surface steeping up with an increase in these elements’ contents. The direction of the steepest “hill climbing” refers to the movement from the area with coordinates “0.3 wt.% C–1.5 wt.% B” to the area with coordinates “0.95 wt.% C–3.5 wt.% B” (for the as-cast state, Figure 11a) and “(0.78–0.85) wt.% C–(2.9–3.1) wt.% B” (for the as-quenched states, Figure 11b,c). Such effect of carbon and boron is explained by a drastic increase in the total volume fraction of hard phases (borocarbides, carboborides), as well as the replacement of ferrite with martensite or “martensite + pearlite/bainite” in the structure of the matrix [39].

The regression equations for the wear rate (×10^−6^ g·mm^−1^·s^−1^) were derived as follows:

*WR_As-cast_* = 4.170 − 1.115*X*_1_ + 0.315*X*_2_ − 0.335*X*_1_*X*_2_ + 0.405*X*_1_^2^ − 1.135*X*_2_^2^ − 0.450*X*_1_^2^*X*_2_^2^ + 0.490*X*_1_*X*_2_^2^ − 0.035*X*_2_*X*_1_^2^, (6)

*WR_1050_* = 2.310 − 1.125*X*_1_ + 0.030*X*_2_ + 0.025*X*_1_*X*_2_ + 0.855*X*_1_^2^ + 0.080*X*_2_^2^ − 0.665*X*_1_^2^*X*_2_^2^ + 0.265*X*_1_*X*_2_^2^ + 0.515*X*_2_*X*_1_^2^, (7)

*WR_1150_* = 0.930 − 0.278*X*_1_ − 0.002*X*_2_ + 0.574*X*_1_*X*_2_ − 0.038*X*_1_^2^ + 0.139*X*_2_^2^ + 0.464*X*_1_^2^*X*_2_^2^ − 0.533*X*_1_*X*_2_^2^ − 0.379*X*_2_*X*_1_^2^, (8)

where Equation (6) was adopted from [39].

The free term of the Equations (6)–(8) decreases from 4.17 for the as-cast state to 0.93 for the 1150 °C-quenching, indicating the general decrease in the wear rate caused by quenching. Negative values of the coefficient at *X*_1_ in all equations indicate that carbon decreases the *WR*. In turn, positive values of the coefficient at *X*_2_ in Equations (6) and (7) highlight that boron increases the *WR* in the as-cast condition and after quenching from 1050 °C. In contrast, under QT = 1150 °C, the coefficient at *X*_2_ changes its sign to negative, meaning that boron increases the wear resistance of the alloys. The different signs of the coefficients for the interactions of variables (quadratic, third-degree, and fourth-degree) indicate the complex non-linear relief of the response surface of the models (6)–(8).

**Figure 12:**
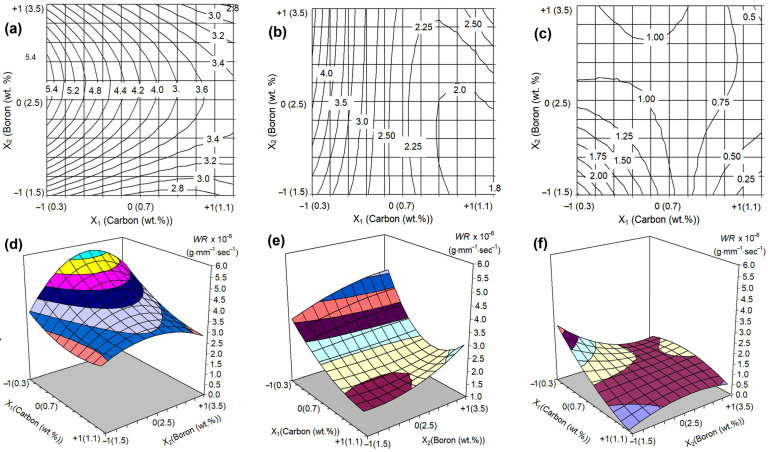
Graphical interpretation of Equations (6)–(8). (**a**–**c**) The response surfaces of a wear rate and (**d**–**f**) its projections on the concentration plot. (**a**,**d**) The as-cast state [42], (**b**,**e**) QT = 1050 °C, (**c**,**f**) QT = 1150 °C.

Figure 12 visualizes Equations (6)–(8), showing the response surfaces and their projections on the concentration plot. It can be seen that in all three cases, the response surface performs a complex relief, the character of which varies depending on the state of the alloys. One can note a general tendency for the surface level to decrease during the transition from the as-cast state to QT = 1150 °C, which corresponds to an increase in the abrasive wear resistance of the alloys. In the as-cast state, carbon continuously reduces the wear rate at any boron content (Figure 12a). Boron, in turn, has a non-monotonous effect, leading to maximum wear at 2.5 wt.% because of an inappropriate microstructure comprising the coarse primary M_2_(B,C)_5_ particles combined with a smaller number of eutectic borocarbide particles. As a result, in the as-cast state, the surface has an inflection (a “hill”) at 2.5 wt.% B, while the minimum points of the surface (the best wear performance) refer to the following alloy compositions: (a) 1.1 wt.% C, 3.5 wt.% B (*WR* of 2.76 × 10^−6^ g·mm^−1^·s^−1^) and (b) 0.86 wt.% C, 1.5 wt.% B (*WR* of 2.67 × 10^−6^ g·mm^−1^·s^−1^).

Under the QT = 1050 °C, the relief of the response surface changes. Boron gradually increases the wear rate at any carbon content. Carbon gradually reduces the *WR* at low boron concentrations and has an opposite effect at 3.5 wt.% B, providing a minimum *WR* at 0.86 wt.% C. The lowest surface point corresponds to the alloy composition of 1.1 wt.% C and 1.7 wt.% B (*WR* of 1.81 × 10^−6^ g·mm^−1^·s^−1^), while the highest point refers to the composition of 0.3 wt.% C and 3.5 wt.% B (*WR* of 4.63 × 10^−6^ g·mm^−1^·s^−1^).

Under the QT = 1150 °C, the pattern of the response surface changed once again. Most of the surface area became almost plane, indicating similar *WR* values for different C/B combinations (within the range of (1.0–1.4) × 10^−6^ g·mm^−1^·s^−1^)). At 1.1 wt.% C and 1.5 wt.% B, the surface has the minimum (0.20 × 10^−6^ g·mm^−1^·s^−1^), while as the concentrations of carbon and boron decrease, the surface steeps up, reaching a maximum (2.79 × 10^−6^ g·mm^−1^·s^−1^) at 0.3 wt.% C and 1.5 wt.% B.

As follows from Figure 2, an increase in the boron concentration to 2.5 wt.% led to the appearance of coarse primary particles in the structure of the alloys. These inclusions had a contradictory effect on wear resistance. On the one hand, they increase the wear resistance due to high hardness (~2400 HV [37]); thus, alumina particles cannot cut them (this leads to a “shadow effect” when the adjacent matrix remains unworn, being shielded by the particle) [42]. On the other hand, the high brittleness of the inclusions leads to their easy cheeping under the abrasive particles’ pressure. The appearance of such inclusions in the as-cast (i.e., with a soft matrix) 2.5 wt.% B alloys accelerate the wear. When the boron content increases to 3.5 wt.%, the number of primary inclusions is increased, thus reducing the preferential wear of the matrix, expanding the “shadow effect”. As a result, the alloys with 3.5 wt.% B show a lower *WR* as compared with those with 2.5 wt.% B. This leads to the non-monotonic effect of boron, with a maximum *WR* value at 2.5 wt.% B. A completely different character of the boron effect is observed after quenching, when hardness sharply increases. In this case, boron advances the wear resistance of the alloys. The QT should be limited to 1150 °C, since this temperature is quite close to the melting point (as follows from the DSC curves). Heating to an even higher temperature (1200 °C) can cause partial melting of the castings, especially taking into account the possible segregation of elements that lower the melting point of the alloy.

Like boron, carbon is involved in the formation of hard phases. However, its effect refers only to Ti-rich carboboride and Fe(Cr)-rich eutectic carboborides M_3_(C,B) and M_7_(C,B)_3_ [37,39]. With that, carbon has a slight effect on the formation of borocarbide M_2_(C,B)_5_; thus, it is not responsible for the solidification of coarse primary inclusions which adversely affect the wear resistance. Also, carbon remarkably contributes to the formation of a wear-resistant matrix with C-rich structural components (acicular martensite, pearlite-bainite conglomerate). These structures originate from austenite, the single-phase domain of which is limited in MCCIs’ phase diagram due to the ferrite-stabilizing effect of Cr, W, Mo, and V. Since carbon belongs to the gamma-stabilizing elements, it expands the austenite domain, thus ensuring the austenite → martensite transformation [64,65]. Therefore, carbon continually reduces the wear rate of the alloy (over its studied concentration range) regardless of the boron content.

### 4.3. “Hardness-Wear Rate” Correlations

**Figure 13:**
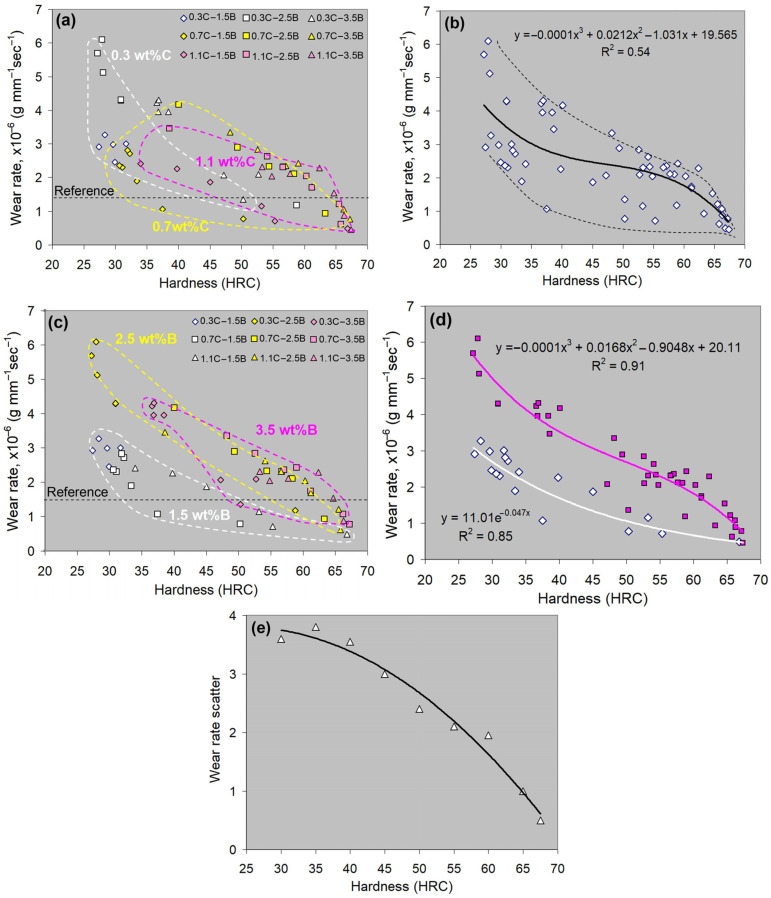
The “Wear rate—Bulk hardness” correlations: (**a**) regarding the carbon content, (**b**) the generalized correlation for different alloys with the scatter limits (dashed lines) and approximating function (solid black line), (**c**) regarding the boron content, (**d**) generalized correlations and approximating functions for the alloys with 1.5 wt.% B (white dots and white line respectively) and 2.5–3.5 wt.%B (pink dots and line respectively). (**e**) Effect of bulk hardness on the maximum scatter in the wear rate for different alloys.

Figure 13 shows the “Hardness-Wear Rate” correlations for various alloys, grouped by the carbon content (Figure 13a) and boron content (Figure 13c). The experimental points on the graphs refer to various alloys both in the as-cast state and heat-treated state (quenching from different temperatures). As follows from Figure 13a, the scatter areas of *WR* values for the carbon-wise groups mostly overlap each other, forming a common band of the values scatter (Figure 13b), which can be described by the following generalized equation:

*WR* = −0.0001·*H*^3^ + 0.021·*H*^2^ − 1.031·*H* + 19.57 (*R*^2^ = 0.55), (9)

where *WR* is measured in (×10^−6^ g·mm^−1^·s^−1^), and *H* is the bulk hardness (HRC).

Equation (9) represents a tendency for the *WR* to decrease with the increasing hardness of the alloys. Carbon has virtually no effect on the pattern of this dependence. In contrast, in boron-wise groups, the scatter area of the 1.5 wt.% B group lies below the areas of the 2.5 wt.% B and 3.5 wt.% B groups (the last two overlap each other) (Figure 13c). Therefore, the “Hardness—*WR*” correlations can be described separately (Figure 13d):

- for 1.5 wt.% B-alloys:

*WR* = 11.01·*exp*(−0.047·*H*) (*R*^2^ = 0.85), (10)

- for 2.5–3.5 wt.% B-alloys:

*WR* = −0.0001·*H*^3^ + 0.017·*H*^2^ − 0.92·*H* + 20.11 (*R*^2^ = 0.91). (11)

Thus, at the same hardness level, the 1.5 wt.%B-alloys show a lower wear rate as compared to the alloys with a higher boron content. This highlights the general advantage of the eutectic structure over the hypereutectic structure (due to the absence of primary M_2_(B,C)_5_ particles). In conclusion, boron affects the correlation “Hardness—*WR*” more significantly as compared to carbon, mostly through its controlling the M_2_(B,C)_5_ formation.

The hybrid MCCIs in the as-cast state significantly vary in structure, resulting in a rather high difference in wear behavior (with a maximum scatter in *WR* values of 3.8 × 10^−6^ g·mm^−1^·s^−1^). Figure 13b presents the general distribution of experimental points (related to both the as-cast and as-quenched alloys) on the “Hardness—*WR*” plot. This figure shows that at similar low hardness values (27–28 HRC), the *WR* values differ maximally by 3.2 × 10^−6^ g·mm^−1^·s^−1^. With increasing hardness, the maximum *WR* scatter decreases to 1.8 × 10^−6^ g·mm^−1^·s^−1^ (at 60 HRC) and 0.32 × 10^−6^ g·mm^−1^·s^−1^ (at 67 HRC) (Figure 13e). Thus, quenching to high hardness (over 60 HRC) allows for leveling the wear resistance of hybrid MCCIs regardless of C and B contents. Such hardness can be reached in MCCIs containing ≥0.7 wt.% C and ≥2.5 wt.% B; also, the alloy 1.1C–1.5B acquired a hardness of 67 HRC after quenching from 1150 °C. Hence, these alloys are interchangeable for abrasive wear applications to a certain extent.

### 4.4. Practical Aspects of Using Hybrid MCCIs

The tribological properties of the hybrid MCCIs were assessed relative to the reference specimen, which was a widely used wear-resistant alloy (high-chromium cast iron) quenched to a maximum hardness (66 HRC). The wear rate of a reference specimen was measured as 1.44 × 10^−6^ g·mm^−1^·s^−1^. The reference *WR* level is indicated in Figure 13a,c by a dashed line. One can see that within the hardness range of <65 HRC, most of the experimental points (75 %) are above the line, i.e., in these cases, the MCCIs were inferior to the reference in wear resistance. At the same time, the minor number of points (which referred to alloys with 0.7–1.1 wt.% C) is below the reference line. This means that even under lower hardness, some hybrid MCCIs performed an advanced wear resistance. Notably, under comparable hardness (>65 HRC), all experimental points are below the reference level, indicating the MCCIs’ advantage in wear resistance over the reference specimen.

**Figure 14:**
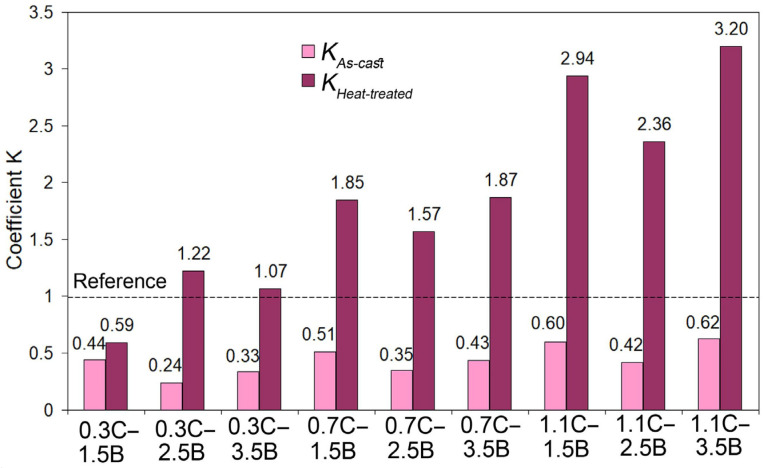
The K values of the hybrid MCCIs in the as-cast and heat-treated states.

The wear behavior of MCCIs was evaluated by the wear resistance coefficient (*K*), which was calculated as

(12) $$K_{i}=\frac{{WR}_{ref}}{{WR}_{i}}$$

where *WR_ref_* and *WR_i_* are the wear rate values for the reference alloy and *i*-experimental alloy, respectively. The *K* values for different MCCIs (in the as-cast and heat-treated states) are depicted in Figure 14 (in the latter case, the highest *K* values are presented).

As seen, in the as-cast state, all experimental MCCIs are inferior to high-Cr cast irons in terms of wear resistance because of the much lower bulk hardness. After quenching, the wear resistance of 0.3 wt.% C-MCCIs is lower or close to the reference (their *K* values are ≤1.22). Taking into account the higher number of alloying elements (i.e., higher cost), this group of MCCIs cannot compete with the reference in terms of practical application. The wear resistance coefficient of 0.7 wt.% C-MCCIs reaches a maximum of 1.57–1.87, which is to be considered a significant advantage over high-Cr cast iron. The best wear behavior is exhibited by the MCCIs containing 1.1 wt.% C. According to the *K* values, they are 2.94 times (alloy 1.1C–1.5B), 2.36 times (alloy 1.1C–2.5B), and 3.20 times (alloy 1.1C–3.5B) more wear-resistant than the reference alloy. The near-threefold advantage in abrasive wear resistance over high-Cr cast iron shows the high potential of hybrid multi-component alloys for industrial applications, even at their higher cost associated with (W,Mo,V)-alloying. The use of alloys is advisable only after heat treatment to maximum hardness (quenching from 1150 °C, tempering at 200 °C). In this case, the increase in the cost of alloys due to alloying with expensive elements can be compensated for by an increase in their durability.

It should be noted that the best wear performance (*K* of about 3.0) was demonstrated under different types of the MCCIs’ structure, specifically near-eutectic (alloy 1.1C–1.5B) or hypereutectic (alloy 1.1C–3.5B). The use of a near-eutectic-type alloy is preferable, since, due to the absence of brittle primary borocarbides in the structure, it potentially has a higher toughness and lesser susceptibility to crack formation under manufacturing and exploitation, which can expand its scope of application. Alloying with Cr, Mo, and W, which are partially dissolved in the matrix, will ensure MCCIs’ high hardenability, thus allowing for the use of air-cooling during quenching to reduce distortion and prevent the cracking in the cast components. The kinetics of austenite transformation in MCCIs should be additionally studied to determine the maximum thickness of castings, which can acquire a martensitic structure under air-cooling.

When assessing the applicability of the material for specific abrasive wear applications, the hardness of abrasive particles should be taken into account. In this study, fairly hard Al_2_O_3_ (1400–2000 HV [66,67]) was used as an abrasive. Al_2_O_3_ is inferior to borocarbide in hybrid MCCIs (~2400 HV [37,38,39]); however, it is of the same order as the chromium carbide M_7_C_3_ in high-Cr cast iron (1300–1800 HV [68]). That is why MCCIs significantly exceed the reference alloy when tested by Al_2_O_3_. Under testing by a softer abrasive (for instance, SiO_2_, ~1000 HV [69]), the wear resistance ratio may change significantly in favor of the reference, since M_7_C_3_ is harder than quartz. At this stage of research, hybrid MCCIs are recommended to be used for the processing of hard sorts of abrasive materials (SiC, Al_2_O_3_, Si_3_N_4_, etc.). Determining the relative wear resistance of the hybrid multi-component cast alloys under testing by a softer abrasive is a challenge for further research.

The results obtained should be considered as a basis for the further improvement of the chemical composition of hybrid multi-component alloys. It became obvious that the high concentration of ferrite-stabilizing alloying elements (W, W, V, Cr, Ti) and the low concentration of gamma-stabilizers (C, Mn) in the MCCIs’ composition lead to the contraction of the domain of austenite existence; it makes achieving high hardness difficult. To reach austenite (which will then turn into hard martensite), it is necessary to heat the alloy to rather high temperatures close to the melting point. This can cause the melting of the castings and promote surface oxidation. Hence, the chemical composition of hybrid MCCIs should be balanced towards the coexistence of αFe and γFe phases at relatively low quenching temperatures. This can be achieved by increasing the contents of gamma-stabilizing elements (Mn, Ni, C); however, a further increase in the carbon concentration is inappropriate, since it can lead to the formation of a hypereutectic structure with coarse carboboride precipitates. In this case, a specific structural state may arise, which will further increase the wear resistance of the alloys. It is about the formation of a heterogeneous matrix combining martensite and retained austenite. With a rational selection of alloying elements and the phase ratio, retained austenite may undergo strain-induced martensitic transformation (SIMT) during wear. It is known that when worn by a hard abrasive, metastable austenite can exhibit an advanced wear resistance, several times higher than that of martensite [70]. This is associated with the high energy consumption by SIMT and the abnormal hardening of austenite [71]. Moreover, austenite, as a ductile phase, may promote the impact toughness of alloys, which will make it possible to operate them under impact loads. The development of hybrid multicomponent alloys with a metastable austenitic matrix is a promising direction for the further research of high-boron alloys intended for tribological applications.

## 5. Conclusions

The effect of the quenching temperature (QT) on the microstructure, bulk hardness, and “Three-Body-Abrasion” wear behavior (in contact with Al_2_O_3_) of hybrid multi-component (wt.%) Fe-(0.3–1.1C)–(1.5–3.5B)-5W-5Mo-5V-10Cr-2.5Ti cast alloys has been studied. The following conclusions were drawn:

- An increase in the QT from 950 to 1150–1200 °C improved most alloys’ hardness and wear resistance. Under low carbon (0.3 wt.%) and/or boron (1.5 wt.%) contents, a noticeable increase in hardness was observed at a QT above 1050 °C, reaching the maximum of 50–59 HRC. In other alloys, the hardness increased proportionally to the QT starting from 950 °C and reached 63–67 HRC. Accordingly, the wear rate decreased by three to six times (relative to the as-cast state). This was due to the formation of a martensitic matrix strengthened by secondary carboboride precipitates. Alloy 0.3C–1.5B retained a ferritic matrix regardless of the QT, and its properties were hardly changed by quenching.
- Carbon increased the wear resistance of MCCIs due to the formation of the carboborides M(C,B), M_7_(C,B)_3_, and M_3_(C,B), as well as by expanding the γ-area, thus contributing to the appearance of the martensitic matrix under the quench cooling. Boron had a versatile effect on wear resistance, which is associated, on the one hand, with the formation of wear-resistant borocarbide phases and, on the other, with the occurrence of coarse primary inclusions that were easily spalled off during wear.
- At any hardness, the alloys with a near-eutectic structure exhibited a higher wear resistance as compared to the hypereutectic ones. This is attributed to a more favorable wear mechanism of the multi-cycle formation/removal of the fine micro-scales. In hypereutectic alloys, the predominant wear mechanism is a spalling of coarse primary borocarbides. With the QT increase, the difference in the wear rate of alloys decreased due to matrix hardness improvement: the hard matrix better resisted wear, preventing the easier exposure and fracture of primary borocarbides.
- The factorial design of experiment 3^2^ was used to optimize the alloy composition. According to the regression models derived, the highest wear resistance is attributed to the MCCI with 1.1 wt.% C and 1.5 wt.% B quenched from 1150 °C. Under the testing, the optimized alloy exhibited a hardness of 67 HRC and a wear rate of 0.88 × 10^−6^ g·mm^−1^·s^−1^. A similar wear resistance was shown by MCCI with 1.1 wt.% C and 3.5 wt.% B (quenching from 1200 °C, 67 HRC, WR of 0.81 × 10^−6^ g·mm^−1^·s^−1^). The above alloys were 2.94 and 3.20 times more wear-resistant than the reference alloy (a 13 wt.% Cr-cast iron, 66 HRC) indicating the high potential of the MCCIs to stand hard-abrasive applications.

## Figures and Tables

**Table 1:** Chemical compositions of the alloys.

Alloy Designation	Coded Levels of the Variables		Content (wt.%)									
	X_1_ (C)	X_2_ (B)	C	B	Si	Mn	W	Mo	V	Cr	Ti	Al
0.3C–1.5B	–1	–1	0.22	1.68	0.95	1.06	5.57	4.80	5.21	10.39	2.55	0.15
0.3C–2.5B	–1	0	0.25	2.70	1.05	1.21	4.68	5.14	5.01	9.85	2.83	0.05
0.3C–3.5B	–1	1	0.30	3.62	1.14	0.88	5.35	5.19	5.35	10.32	2.78	0.08
0.7C–1.5B	0	–1	0.77	1.62	1.12	1.16	5.84	5.38	4.97	10.45	2.93	0.05
0.7C–2.5B	0	0	0.72	2.75	1.10	0.90	5.05	5.57	5.78	10.35	2.60	0.04
0.7C–3.5B	0	1	0.70	3.61	1.18	1.07	4.67	4.63	5.40	10.21	2.71	0.08
1.1C–1.5B	1	–1	1.20	1.59	1.07	1.10	5.42	4.48	5.37	10.41	2.38	0.10
1.1C–2.5B	1	0	1.11	2.73	1.10	1.07	4.85	4.69	5.26	10.36	2.43	0.14
1.1C–3.5B	1	1	1.13	3.57	1.06	1.03	4.50	4.08	4.79	9.94	2.39	0.11

**Table 2:** The temperature ranges of the endothermic effects revealed on the DSC curves (Figure 1).

Alloy	The Temperature Range (°C) of the Endothermic Effects			
	Effect #1	Effect #2	Effect #3	Melting
0.3C–1.5B	593–757	–	979–1200	>1200
0.3C–2.5B	598–776	–	1020–1178	>1178
0.3C–3.5B	581–780	–	1023–1170	>1170
0.7C–1.5B	578–747	–	1065–1152	>1152
0.7C–2.5B	662–790	880–974	1062–1146	>1146
0.7C–3.5B	622–761	894–1048	–	>1120
1.1C–1.5B	635–741	–	1060–1160	>1160
1.1C–2.5B	677–771	883–960	1067–1135	>1135
1.1C–3.5B	635–767	956–1052	–	>1113

## Data Availability

The original contributions presented in the study are included in the article, further inquiries can be directed to the corresponding author.
